# Population Trends and Individual Fluidity of Sexual Identity Among Stockholm County Residents

**DOI:** 10.1001/jamanetworkopen.2024.47627

**Published:** 2024-12-04

**Authors:** Willi Zhang, Per Tynelius, Maya B. Mathur, Matteo Quartagno, Gunnar Brandén, Fredrik Liljeros, Kyriaki Kosidou

**Affiliations:** 1 Department of Global Public Health, Karolinska Institutet, Stockholm, Sweden; 2 Centre for Epidemiology and Community Medicine, Region Stockholm, Stockholm, Sweden; 3 Quantitative Sciences Unit, Stanford University, Palo Alto, California; 4 MRC Clinical Trials Unit, University College London, London, United Kingdom; 5 Department of Sociology, Stockholm University, Stockholm, Sweden

## Abstract

This survey study examines population trends in sexual identity and patterns of individual sexual identity fluidity in Stockholm County, Sweden, from 2010 to 2021.

## Introduction

Sexual identity, which is linked to experiences of disadvantage and discrimination, is crucial in equality monitoring. In Sweden, legal recognition of same-sex unions was introduced in 1995 and gender-neutral marriage legislation in 2009.^1^ However, it remains unknown how population trends in sexual identity have evolved in Sweden. Furthermore, sexual identity can be fluid, particularly among lesbian, gay, bisexual, transgender, or queer (LGBTQ+) individuals, complicating the estimation of LGBTQ+ population sizes.^2^ We examined population trends in sexual identity and patterns of individual sexual identity fluidity in Stockholm County from 2010 to 2021.

## Methods

This cohort study was approved by the Stockholm Regional Ethical Review Board. Written informed consent was obtained from all individual participants included in the study. The study followed the American Association for Public Opinion Research (AAPOR) reporting guideline.

We analyzed 5 population-representative, prospective cohorts from the Stockholm Public Health Cohort, initiated from 2002 to 2021 and followed up until 2021 (eMethods in Supplement 1). Sexual identity was self-reported in 2010, 2014, and 2021 (eTable in Supplement 1). To examine patterns of individual sexual identity fluidity, we pooled data from the 2002, 2006, and 2010 cohorts (eFigures 1-3 in Supplement 1). Calibrated weights were applied to account for unequal selection probabilities and unit nonresponse,^3^ and 2-level multivariate normal imputation for item nonresponse.^4^ Data were analyzed from June 2023 to September 2024 using R version 4.3.1 (R Project for Statistical Computing).

## Results

**Figure 1.**
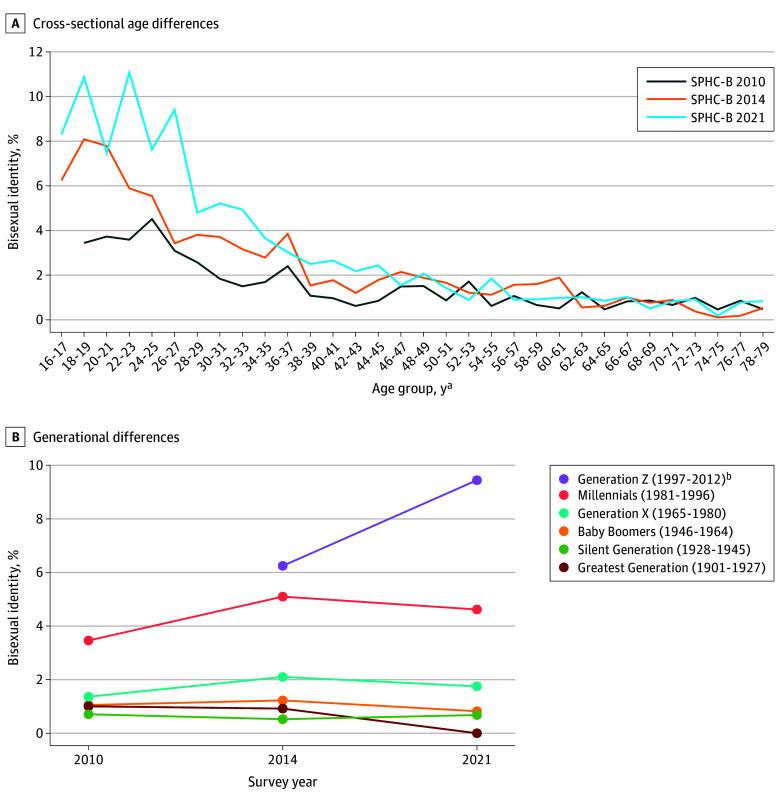
Age, Generation, and Period Effects and Bisexual Identity The results were based on multiple imputation analyses, incorporating calibrated weights to account for the stratified random sampling design and the potential unit nonresponse bias. SPHC-B indicates Stockholm Public Health Cohort baseline survey. ^a^Minimum age was 18 years in the 2010 survey, and 16 years in the 2014 and 2021 surveys. ^b^No participants were from Generation Z in the 2010 survey.

The study included 98 317 of 141 972 (69.3%) participants (mean [SD] age, 50.5 [17.5] years; 54 553 [55.5%] females). Bisexual identity increased from 1.6% (95% CI, 1.4%-1.8%) in 2010 to 2.5% (95% CI, 2.2%-2.9%) in 2014 and 3.1% (95% CI, 2.8%-3.4%) in 2021, while homosexual identity ranged from 1.7% (95% CI, 1.5%-1.9%) in 2010 to 2.0% (95% CI, 1.7%-2.2%) in 2021. Bisexual identity decreased with increasing age and increased with younger generations in all 3 survey years (Figure 1). In 2021, homosexual and bisexual identity was 12.0% (95% CI, 10.3%-13.7%) in Generation Z (ie, born between 1997 and 2012), 7.8% (95% CI, 6.9%-8.6%) in Millennials (ie, born between 1981 and 1996), and 3.4% (95% CI, 2.8%-3.9%) in Generation X (ie, born between 1965 and 1980).

**Figure 2.**
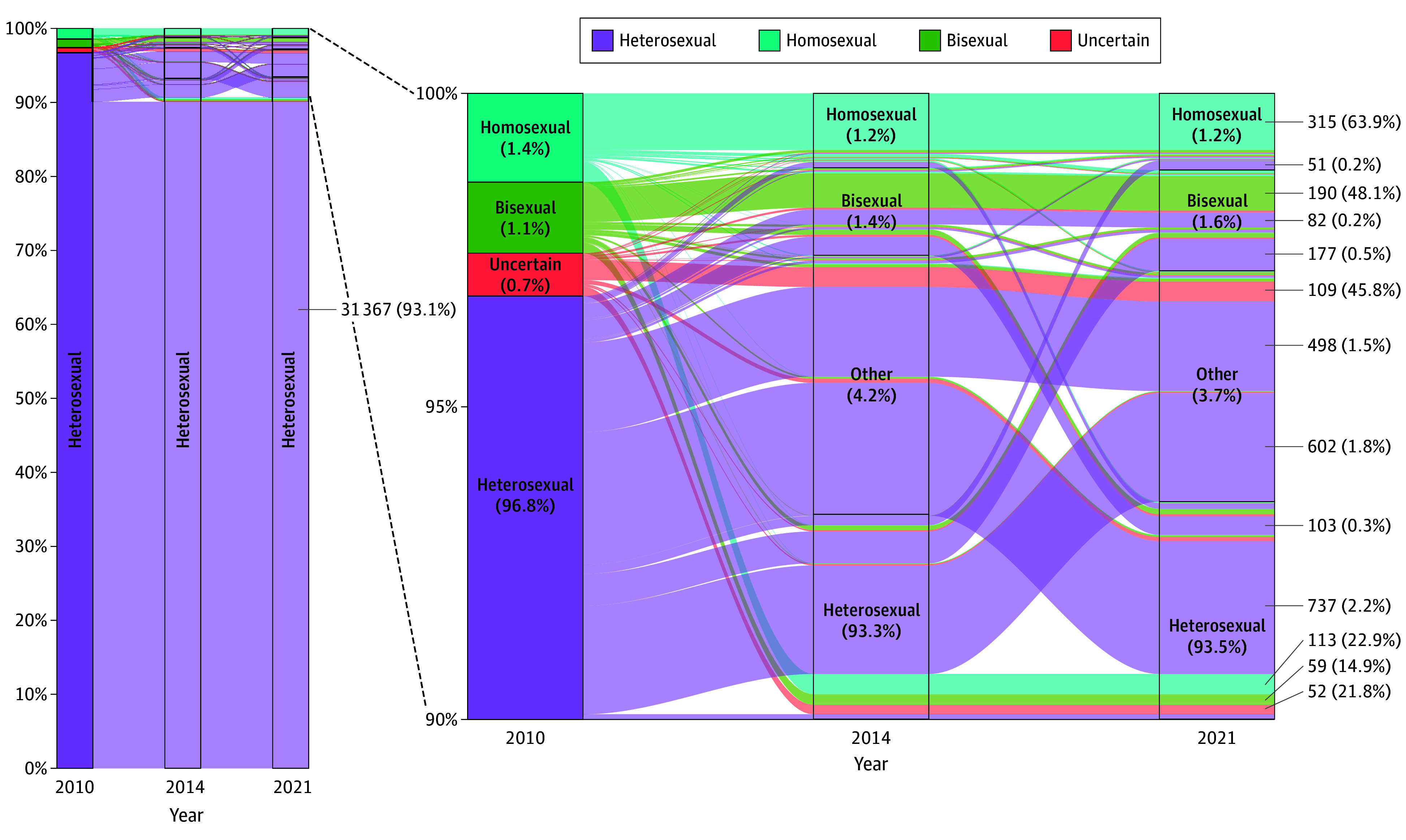
Individual Fluidity of Sexual Identity, 2010-2021 Among all participants in the 2002, 2006, and 2010 cohorts who provided data on sexual identity in 2010, 2014, and 2021 (N = 34 815; eFigure 2 in Supplement 1). Other represents none of the above. The percentages within each box denote the proportions of each sexual identity in the respective survey years. In certain columns, the percentages may not total 100% due to rounding. The numbers and percentages on the right side of the plot illustrate the counts and proportions of individuals within each sexual identity group in 2010 who either retained their sexual identity or reported a change from 2010 to 2021.

Among the pooled cohort, bisexual identity increased from 1.1% in 2010 to 1.4% in 2014 and 1.6% in 2021 (Figure 2). For individuals aged 18 to 29 years, bisexual identity increased from 3.9% in 2010 to 4.9% in 2014 and 5.4% in 2021. For individuals aged 30 to 44 years, bisexual identity increased from 1.6% in 2010 to 2.1% in 2014 and 2.8% in 2021. In 2021, 45.8% to 50.6% of those identifying as bisexual had previously identified as heterosexual in 2010.

Overall, 15.7% (95% CI, 14.2%-17.2%) changed their identity at least once between 2010 and 2021 in the 2010 cohort, and 9.6% (95% CI, 6.4%-12.7%) changed their identity between 2014 and 2021 in the 2014 cohort.

## Discussion

With the relatively stable accepting climate toward LGBTQ+ people in Sweden since 2010,^1^ it seems reasonable to assume minimal period effects on sexual identity from 2010 to 2021. Thus, the continuous increase in bisexual identity among the younger age groups during the 12-year follow-up may suggest age effects, where bisexual self-identification increases with age among younger people. Notably, this increase was primarily driven by younger individuals transitioning from heterosexual to bisexual identity. However, this was contradicted by the decreasing trend seen in the cross-sectional age differences. This contradiction seemed to be due to generation effects, where older generations were much less likely to identify as bisexual than younger generations, thereby reversing the increasing trend with age among the younger age groups. The bisexual group has become the largest sexual minority group in Stockholm County since 2014, and in the US since 2016 where bisexual identity reached 15.3% among Generation Z in 2023.^5,6^ Given the strong generation effects, we expect that the bisexual population will continue to grow in Sweden and potentially in other high-income countries. Future studies are warranted to investigate the substantial health disadvantages faced by the bisexual individuals and whether transitions between identities represent a critical time window for health. Limitations include potential measurement bias of sexual identity and the application of social generations from the US context to the Swedish context.

## Notes

Supplement 1.
eMethods.
**eTable.** Measurement of Sexual Identity in the Stockholm Public Health Cohort **eFigure 1.** Overview of the Baseline and Follow-Up Surveys in the Stockholm Public Health Cohort from 2002 to 2021 **eFigure 2.** Study Samples for Individual Fluidity of Sexual Identity **eFigure 3.** Flow Diagram of Selection of Participants in the Stockholm Public Health Cohort

Supplement 2.
Data Sharing Statement
