# Changes to Author Name and Email

**DOI:** 10.1001/jamanetworkopen.2024.59848

**Published:** 2025-01-09

In the Research Letter titled “Population Trends and Individual Fluidity of Sexual Identity Among Stockholm County Residents,”^1^ published December 4, 2024, the author, previously listed as Guoqiang Zhang, has changed his name to Willi Zhang. The corresponding author’s email address has also been updated to willi.zhang@ki.se. This article has been corrected.^1^
