# Supplementary material for: Population Trends and Individual Fluidity of Sexual Identity Among Stockholm County Residents
Source: JAMA Netw Open. 2024 Dec 4;7(12):e2447627. doi: 10.1001/jamanetworkopen.2024.47627 (PMC11618470; doi:10.1001/jamanetworkopen.2024.47627)
Supplement: Supplement 1. — eMethods. eTable. Measurement of Sexual Identity in the Stockholm Public Health Cohort eFigure 1. Overview of the Baseline and Follow-Up Surveys in the Stockholm Public Health Cohort from 2002 to 2021 eFigure 2. Study Samples for Individual Fluidity of Sexual Identity eFigure 3. Flow Diagram of Selection of Participants in the Stockholm Public Health Cohort [file jamanetwopen-e2447627-s001.pdf]

Supplemental Online Content

Zhang W, Tynelius P, Mathur MB, et al. Population trends and individual fluidity of sexual identity among Stockholm county residents. *JAMA Netw Open*. 2024;7(12):e2447627. doi:10.1001/jamanetworkopen.2024.47627

eMethods.
eTable. Measurement of Sexual Identity in the Stockholm Public Health Cohort
eFigure 1. Overview of the Baseline and Follow-Up Surveys in the Stockholm Public Health Cohort from 2002 to 2021
eFigure 2. Study Samples for Individual Fluidity of Sexual Identity
eFigure 3. Flow Diagram of Selection of Participants in the Stockholm Public Health Cohort
eReferences

This supplemental material has been provided by the authors to give readers additional information about their work.

© 2024 Zhang W et al. *JAMA Network Open.*

eMethods.

1. Study Population

The Stockholm Public Health Cohort (SPHC) is a prospective, population-based cohort established in Stockholm County in Sweden, which has been described in detail elsewhere.[1] So far, SPHC comprises five baseline surveys conducted in 2002, 2006, 2010, 2014, and 2021, along with multiple follow-up surveys. eFigure 1 presents an overview of the baseline and follow-up surveys. For the baseline surveys, based on the Swedish Total Population Register (TPR) from Statistics Sweden,[2] area-stratified random samples were drawn from the population of Stockholm County aged 18–84 years in the 2002 and 2006 surveys (in the 2002 survey, the stratification was based on both area and sex), aged 18 years or older in the 2010 survey, and aged 16 years or older in the 2014 and 2021 surveys. There were 38 to 43 municipalities and urban districts, and more than 1,000 individuals were randomly selected per municipality/urban district, resulting in a total sample size of around 50,000 individuals in each survey. In the 2002, 2006, and 2021 surveys, the entire population in each year served as the sampling frame. A total of 999 individuals participated in both the 2002 and 2006 surveys. In the 2010 and 2014 surveys, the sampling frames were constructed after excluding individuals who had participated in earlier surveys. The approximate sampling frames were 1.4 million in 2002, 1.5 million in 2006, 1.6 million in 2010, and 1.8 million in 2014 and 2021. The respective SPHC baseline surveys are referred to as “SPHC-B + YEAR”; for example, SPHC-B 2010 represents the baseline survey in 2010.

Data were collected using postal questionnaires in SPHC-B 2002, and both postal and web-based questionnaires in SPHC-B 2006, 2010, and 2014. In SPHC-B 2021, data collection was complemented with a smartphone application called Hälsometern.[3] The questionnaires included assessments of physical and mental health, lifestyle, and social characteristics, publicly available here. In Sweden, each individual registered as a resident is assigned a unique personal identity number, thereby enabling linkage to the Swedish national and regional registers.[4] Therefore, the self-reported questionnaire data in SPHC surveys were supplemented with information from a range of national and regional health and administrative registers.

Participants were resurveyed consecutively according to the new waves of data collection, as illustrated in eFigure 1. Specifically, participants in SPHC-B 2002 were resurveyed in 2007, 2010, 2014, and 2021, those in SPHC-B 2006 were resurveyed in 2010, 2014, and 2021, those in SPHC-B 2010 were resurveyed in 2014 and 2021, and those in SPHC-B 2014 were resurveyed in 2021. The follow-up surveys are referred to as “SPHC-F + YEAR”; for example, SPHC-F 2014 represents the follow-up surveys conducted in 2014. “SPHC + YEAR” encompasses the baseline and follow-up surveys for a given cohort; for example, SPHC 2010 represents the cohort including the baseline survey in 2010 and the subsequent follow-up surveys in 2014 and 2021.

To analyze population trends in sexual identity, the study samples included SPHC-B 2010, 2014, and 2021 (eFigure 1). For individual fluidity of sexual identity, the study samples are depicted in eFigure 2. eFigure 3 illustrates the selection of participants in our study.

© 2024 Zhang W et al. *JAMA Network Open.*

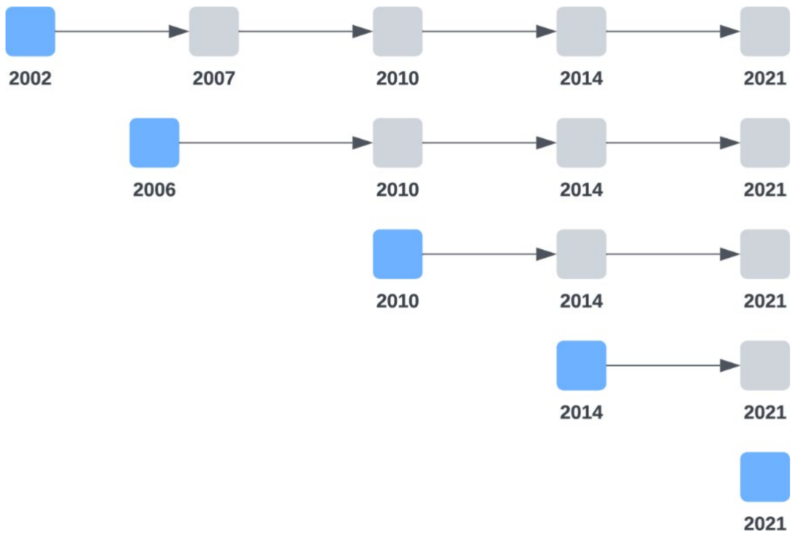

eFigure 1. Overview of the Baseline and Follow-Up Surveys in the Stockholm Public Health Cohort from 2002 to 2021

The baseline surveys are denoted by blue-colored boxes, each labeled with the respective calendar year. Participants from each baseline survey were followed prospectively through to 2021, which is indicated by grey-colored boxes alongside their corresponding years.

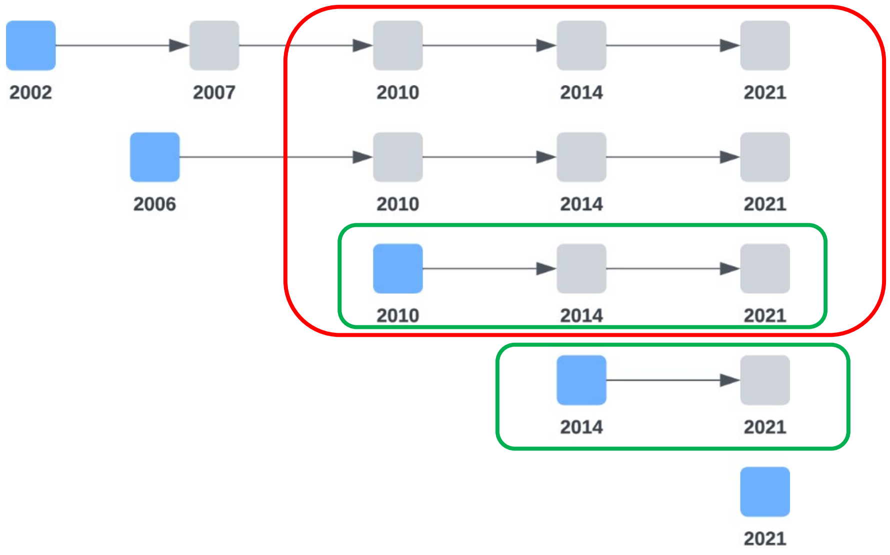

eFigure 2. Study Samples for Individual Fluidity of Sexual Identity

The baseline surveys are denoted by blue-colored boxes, each labeled with the respective calendar year. Participants from each baseline survey were followed prospectively through to 2021, which is indicated by grey-colored boxes alongside their corresponding years. To examine patterns of sexual identity fluidity, the study sample, highlighted in red, consisted of individuals who provided data on sexual identity in 2010, 2014, *and* 2021. Note that among these individuals, 999 participated in both the SPHC-B 2002 and 2006 surveys; thus, their follow-up data were included only once to avoid duplication. To estimate proportions of change in sexual identity, the study sample included two subsamples, marked in green.

© 2024 Zhang W et al. *JAMA Network Open.*

2. Sexual Identity

Data on sexual identity have been collected since 2010, specifically in SPHC-B and SPHC-F surveys from 2010 to 2021. The survey question was *"How do you define your sexual orientation?"* with four response options: *"heterosexual"*, *"homosexual"*, *"bisexual"*, or *"uncertain"* or *"none of the above"* (see eTable). Participants were categorized into one of the following four groups: heterosexual, homosexual, bisexual, or other (i.e., *"uncertain"* or *"none of the above"*). Change in sexual identity (yes or no) was determined by comparing sexual identities reported in at least two survey years.

Notes: **Sexual identity** refers to *"personally selected, socially and historically bound labels attached to the perceptions and meanings individuals have about their sexuality"*.[5] **Sexual attraction** refers to *"attraction toward one sex or the desire to have sexual relations or to be in a primary loving, sexual relationship with one or both sexes"*.[5] **Sexual behavior** refers to *"any mutually voluntary activity with another person that involves genital contact and sexual excitement or arousal, that is, feeling really turned on, even if intercourse or orgasm did not occur"*.[6]

eTable. Measurement of Sexual Identity in the Stockholm Public Health Cohort

Survey year	Response options
2010	Heterosexual; homosexual; bisexual; *uncertain*
2014	Heterosexual; homosexual; bisexual; *none* of the above
2021	Heterosexual; homosexual; bisexual; *none* of the above

Abbreviations: SPHC-B/F, Stockholm Public Health Cohort baseline or follow-up survey.

© 2024 Zhang W et al. *JAMA Network Open.*

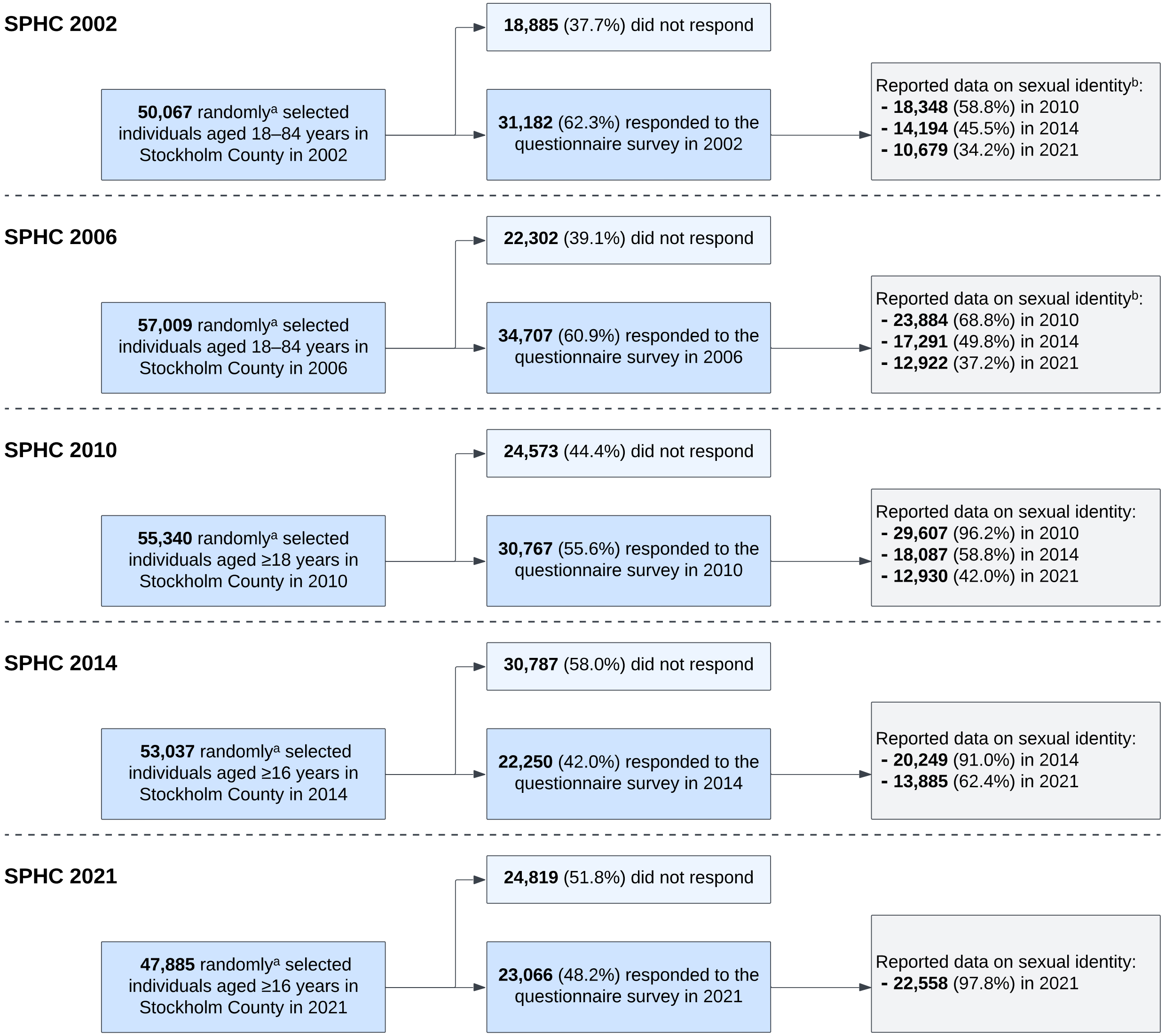

eFigure 3. Flow Diagram of Selection of Participants in the Stockholm Public Health Cohort

Abbreviations: SPHC, Stockholm Public Health Cohort.

[a] Area-stratified random samples were drawn, with 88 sampling strata in SPHC 2002, 43 strata in SPHC 2006, 39 strata in SPHC 2010 and 2014, and 38 strata in SPHC 2021. In SPHC 2002, 2006, and 2021, the entire population in each year served as the sampling frame. A total of 999 individuals participated in both SPHC 2002 and 2006. For SPHC 2010 and 2014, the sampling frames were constructed after excluding individuals who had participated in earlier surveys.

[b] Data on sexual identity started to be collected in 2010.

© 2024 Zhang W et al. *JAMA Network Open.*

3. Statistical Analyses

3.1. Proportions of sexual identities

3.1.1. Complete-case analysis

In these analyses, we assumed that the missing mechanism of sexual identity was **Missing Completely At Random** (MCAR) for both unit and item non-response within each sampling stratum. That is, within each stratum, the responding sample with complete data was *assumed* to be a representative subset of the source population. Thus, the analyses were restricted to individuals with complete data on sexual identity.[7] However, complete-case analysis causes loss of information and may lead to biased estimates.[8]

We utilized the 'survey' package,[9] a statistical tool designed for complex survey data analysis, to estimate the proportions of different sexual identities in Stockholm County and among various age and generation groups.[10] Sampling weights (i.e., the inverse of the probability of selection into the sample) were used to account for unequal selection probabilities. The analyses were performed separately for SPHC-B 2010, 2014, and 2021.

3.1.2. Survey weights and multiple imputation analysis

To deal with the missing data, we combined **survey weights** (SWs) and **multiple imputation** (MI) (referred to as SWs & MI).[11] SWs accounts for potential unit non-response bias, while MI compensates for potential item non-response bias, with both methods assuming **Missing At Random** (MAR).[11-13] Briefly, missing values in *responding* individuals are multiply imputed and then each imputed dataset is analyzed using SWs.[9-11] It has been suggested that SWs & MI can offer certain advantages over using either SWs or MI alone.[11,14] For example, in SWs alone, individuals with partially observed data have to be excluded from the weighted analysis, while SWs & MI allows for imputation of the missing values in these individuals and includes them in the analysis.[7,11] In MI alone, when there is significant amount of missing data, the potential for a misspecified imputation model may lead to considerable bias (see Seaman et al 2012[11] for further discussion).

Specifically, in our study, we applied **calibrated weights** to account for unequal selection probabilities and unit non-response,[15-17] and **two-level multivariate normal imputation** (MVNI) for item non-response.[13,18] In the two-level MVNI, calibrated weights were stratified to define level-two clusters (n = 25).[19] We used MVNI with the latent normal variables approach to impute missing values in categorical variables.[20] Given different proportions of missingness, we imputed 80 datasets for SPHC 2010, 60 for SPHC 2014, and 20 for SPHC-B 2021.[21]

3.2. Individual fluidity of sexual identity

We examined the temporal evolution of sexual identity from 2010 to 2021, among participants from SPHC 2002, 2006, and 2010 who reported their sexual identity in all three survey years (2010, 2014, and 2021) (eFigure 2: indicated by red box). We

© 2024 Zhang W et al. *JAMA Network Open.*

utilized an alluvial diagram to illustrate the shifts in sexual identity from 2010 to 2021, providing insights into the longitudinal patterns and potential fluidity of sexual identity.

We used the 'survey' package[9] to estimate the proportion of change in sexual identity in the general population. The analyses were conducted separately for SPHC 2010 and 2014 cohorts (eFigure 2: indicated by green boxes).

3.2.1. Complete-case analysis

See Section 3.1.1. To estimate the proportion of change in sexual identity, the analyses were restricted to individuals with complete data on change in sexual identity.[7] The underlying assumption was that the missing mechanism was MCAR for both unit and item non-response within each sampling stratum. Sampling weights were applied to adjust for unequal selection probabilities in SPHC 2010 and 2014 cohorts.

3.2.2. Survey weights and multiple imputation analysis

See Section 3.1.2. To estimate the proportion of change in sexual identity, SWs & MI was conducted for SPHC 2010 and 2014 cohorts.

4. Reproducibility

We adopted an open reproducible research workflow.[22] All statistical analyses were conducted using an open-source software R, version 4.3.1.[23] The R scripts are publicly available at GitHub. The datasets used in the analyses were prepared independently by an investigator (Per Tynelius).

eReferences

1. Svensson AC, Fredlund P, Laflamme L, et al. Cohort profile: the Stockholm Public Health Cohort. *Int J Epidemiol*. 2013;42(5):1263-1272.
2. Ludvigsson JF, Almqvist C, Bonamy AK, et al. Registers of the Swedish total population and their use in medical research. *Eur J Epidemiol*. 2016;31(2):125-136.
3. Hälsometern. https://www.ces.regionstockholm.se/halsometern.
4. Ludvigsson JF, Otterblad-Olausson P, Pettersson BU, Ekbom A. The Swedish personal identity number: possibilities and pitfalls in healthcare and medical research. *Eur J Epidemiol*. 2009;24(11):659-667.
5. Savin-Williams RC. Who's gay? Does it matter? *Curr Dir Psychol Sci*. 2006;15(1):40-44.
6. Laumann EO, Gagnon JH, Michael RT, Michaels S. *The Social Organization of Sexuality: Sexual Practices in the United States*. The University of Chicago Press; 1994.
7. Little RJ, Carpenter JR, Lee KJ. A comparison of three popular methods for handling missing data: complete-case analysis, inverse probability weighting, and multiple imputation. *Sociol Methods Res*. 2024;53(3):1105-1135.
8. White IR, Carlin JB. Bias and efficiency of multiple imputation compared with complete-case analysis for missing covariate values. *Stat Med*. 2010;29(28):2920-2931.
9. Lumley T. survey: analysis of complex survey samples. R package version 4.2, 2023.
10. Lumley T. Chapter 2: Simple and stratified sampling. In: Lumley T. *Complex Surveys: A Guide to Analysis Using R*. John Wiley & Sons; 2010.
11. Seaman SR, White IR, Copas AJ, Li L. Combining multiple imputation and inverse-probability weighting. *Biometrics*. 2012;68(1):129-137.
12. Kalton G, Flores-Cervantes I. Weighting methods. *J Off Stat*. 2003;19(2):81-97.
13. Quartagno M, Grund S, Carpenter J. Jomo: a flexible package for two-level joint modelling multiple imputation. *R J*. 2019;11(2):205-228.
14. Kalpourtzi N, Carpenter JR, Touloumi G. Handling missing values in surveys with complex study design: a simulation study. *J Surv Stat Methodol*. 2024;12(1):105-129.
15. Szymkowiak M. Weighting and estimation: calibration. In: *Memobust Handbook on Methodology of Modern Business Statistics*. Eurostat; 2014.
16. Lundström S, Särndal C. Calibration as a standard method for treatment of nonresponse. *J Off Stat*. 1999;15(2):305-327.
17. Lundström S, Särndal C. *Estimation in the Presence of Nonresponse and Frame Imperfections*. Statistics Sweden; 2001. https://share.scb.se/ov9993/data/publikationer/statistik/_publikationer/ov9999_2000i02_br_x97öp0103.pdf. Accessed June 22, 2024.
18. Quartagno M, Carpenter J, Goldstein H. Multiple imputation with survey weights: a multilevel approach. *J Surv Stat Methodol*. 2020;8(5):965-989.
19. Audigier V, White IR, Jolani S, et al. Multiple imputation for multilevel data with continuous and binary variables. *Stat Sci*. 2018;33(2):160-183.
20. Quartagno M, Carpenter JR. Multiple imputation for discrete data: evaluation of the joint latent normal model. *Biom J*. 2019;61(4):1003-1019.
21. White IR, Royston P, Wood AM. Multiple imputation using chained equations: issues and guidance for practice. *Stat Med*. 2011;30(4):377-399.
22. Mathur MB, Fox MP. Toward open and reproducible epidemiology. *Am J Epidemiol*. 2023;192(4):658-664.

© 2024 Zhang W et al. *JAMA Network Open.*

23. R Core Team. R: a language and environment for statistical computing. R Foundation for Statistical Computing, Vienna, Austria, 2023. https://www.R-project.org/.

© 2024 Zhang W et al. *JAMA Network Open.*
