# Supplementary material for: Population Trends and Individual Fluidity of Sexual Identity Among Stockholm County Residents
Source: JAMA Netw Open. 2024 Dec 4;7(12):e2447627. doi: 10.1001/jamanetworkopen.2024.47627 (PMC11618470; doi:10.1001/jamanetworkopen.2024.47627)
Supplement: Supplement 2. — Data Sharing Statement [file jamanetwopen-e2447627-s002.pdf]

Data Sharing Statement

Zhang. Population Trends and Individual Fluidity of Sexual Identity Among Stockholm County Residents. *JAMA Netw Open*. Published December 04, 2024.
doi:10.1001/jamanetworkopen.2024.47627

Data
Data available: No

Additional Information
Explanation for why data not available: The datasets generated and/or analyzed during the current study are not publicly available due to privacy and ethical reasons, but can be accessed after approved ethics application to the Steering Committee of the Stockholm Public Health Cohort (https://www.ces.regionstockholm.se/projekt-och-uppdrag/halsa-stockholm/SPHC-data/). The R scripts are publicly available at GitHub (https://github.com/willizhang/Population-Trends-and-Individual-Fluidity-of-Sexual-Identity-in-Stockholm-County-2010-to-2021).
